# Supplementary material for: Conditional chemoconnectomics (cCCTomics) as a strategy for efficient and conditional targeting of chemical transmission
Source: eLife. 2024 Apr 30;12:RP91927. doi: 10.7554/eLife.91927 (PMC11060718; doi:10.7554/eLife.91927)
Supplement: Figure 3—source data 3. [file elife-91927-fig3-data3.zip › Figure 3 source data3 part 2.zip!/Figure 3-Source data 3-part2/1060f2-1-CRISPResso_Report_gl5umwyq.zip!/CRISPResso_on_1060f2_3/9.Alleles_frequency_table_around_sgRNA_gcccacttcctggagtatcg.pdf]

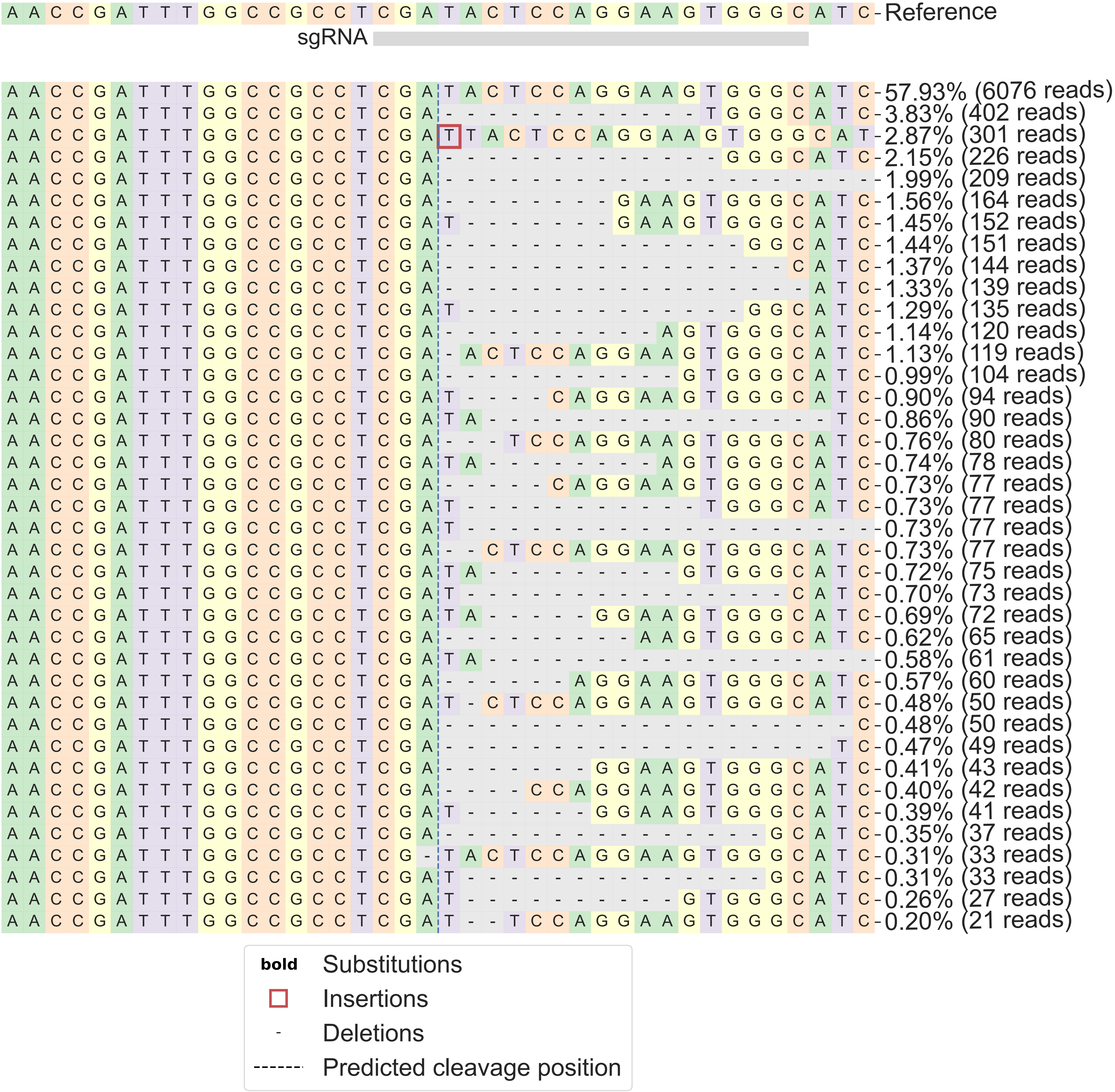

A A C C G A T T T G G C C G C C T C G A T A C T C C A G G A A G T G G G C A T C Reference
sgRNA
57.93% (6076 reads)
3.83% (402 reads)
2.87% (301 reads)
2.15% (226 reads)
1.99% (209 reads)
1.56% (164 reads)
1.45% (152 reads)
1.44% (151 reads)
1.37% (144 reads)
1.33% (139 reads)
1.29% (135 reads)
1.14% (120 reads)
1.13% (119 reads)
0.99% (104 reads)
0.90% (94 reads)
0.86% (90 reads)
0.76% (80 reads)
0.74% (78 reads)
0.73% (77 reads)
0.73% (77 reads)
0.73% (77 reads)
0.73% (77 reads)
0.72% (75 reads)
0.70% (73 reads)
0.69% (72 reads)
0.62% (65 reads)
0.58% (61 reads)
0.57% (60 reads)
0.48% (50 reads)
0.48% (50 reads)
0.47% (49 reads)
0.41% (43 reads)
0.40% (42 reads)
0.39% (41 reads)
0.35% (37 reads)
0.31% (33 reads)
0.31% (33 reads)
0.26% (27 reads)
0.20% (21 reads)
bold Substitutions
Insertions
Deletions
Predicted cleavage position
